# Does Self-Reported Trait Mindfulness Contribute to Reducing Perceived Stress in Women Who Practice Yoga and Are Physically Active?

**DOI:** 10.3390/bs14090772

**Published:** 2024-09-02

**Authors:** Natalia Cavour-Więcławek, Aleksandra M. Rogowska

**Affiliations:** Institute of Psychology, University of Opole, 45-052 Opole, Poland

**Keywords:** mindfulness, perceived stress, physical activity, women’s health, yoga practice

## Abstract

(1) Background: This study aimed to investigate whether yoga practice and physical activity level play an essential role in trait mindfulness and perceived stress reduction. Moreover, the study examined the differences in trait mindfulness and perceived stress between women who practiced yoga and those who engaged in other physical activities or were physically inactive, taking into account the time spent on physical activity in accordance with the World Health Organization recommendations. (2) Methods: A sample of 201 women participated in a cross-sectional online-based study, including 96 yoga practitioners and 105 non-practitioners of yoga (including physically active and inactive individuals). The average age of the participants was 36 years (range, 18–72 years; M = 36.19, SD = 11.64). Respondents completed the Mindful Attention Awareness Scale (MAAS) and the Perceived Stress Scale (PSS-10). (3) Results: Women who practiced yoga and were physically active had a significantly higher level of trait mindfulness and lower perceived stress levels than women who did not practice yoga and were physically inactive. An indirect effect of physical activity on perceived stress through mindfulness was shown only for women practicing yoga for at least 150 min per week. (4) Conclusions: This study revealed the importance of frequent yoga practice in reducing perceived stress and improving mindfulness traits. These findings may serve as a basis for implementing preventive actions in women experiencing high levels of everyday stress.

## 1. Introduction

Presently, there is an increased interest in the concept of mindfulness [1,2,3] The development of therapeutic measures based on mindfulness practice in the 1970s began the psychological research on mindfulness [4]. Two significant perspectives can be discerned by examining the definition of mindfulness [5]. The first one points to the intentional practice of mindfulness, which involves directing one’s attention to what is happening in the present moment while refraining from judging the content of that experience [6]. The other perspective looks at mindfulness as a trait that encompasses an individual’s general tendency to pay attention to the present moment, independent of their experience with meditative practices [5,7,8,9]. The opposite of this trait could involve ruminations, excessive focus on the past and future, and multitasking [7].

Trait mindfulness is distinct from the Big Five traits, although it seems to be more closely related negatively to neuroticism and positively to conscientiousness [7,8,10,11,12,13,14]. As noted by Kiken et al. [15], trait mindfulness can be developed with the use of a range of interventions based on mindfulness practices, which brings about positive implications for mental health. Among individuals undergoing such interventions, a significant reduction in the symptoms of depression and anxiety, as well as an improvement in quality of life have been noted in both clinical and non-clinical samples [3,5,16,17,18,19]. Individuals with higher levels of trait mindfulness better cope with stress and show higher levels of subjective well-being [7,10,19,20].

Nowadays, people face various challenges, which often trigger stress [21]. Stress is a common experience that arises in response to challenging or difficult situations, and it typically elicits a sense of worry or mental tension [22]. Stress impedes relaxation and concentration, heightening negative emotions such as anxiety and irritability. Prolonged stress may lead to headaches, body aches, stomach upset, sleep disturbances, appetite loss, or altered eating habits. It can exacerbate existing health issues and elevate the use of alcohol, tobacco, and other substances, potentially aggravating mental health conditions such as anxiety and depression. The individual manner of coping with stress has a profound influence on overall health and well-being [22]. Recent events such as the COVID-19 pandemic and the outbreak of war in Ukraine have left people feeling anxious and uncertain about their health and future [23]. A systematic review has shown that among students, both anxiety and stress levels increased during the pandemic and as a result of the outbreak of war [23]. Women were characterized by higher scores on both scales compared to men [23]. Trait mindfulness predicted lower stress reactivity among men exposed to a stressor in the form of carbon dioxide inhalation [20]. Prolonged high levels of perceived stress can bring adverse effects on the health of individuals and can negatively affect their well-being [24,25,26,27].

The World Health Organization recommends that adults aged 18–64 engage in at least 150 min of physical activity per week [28]. Physical activity can be an effective intervention for reducing depression, anxiety, and the levels of perceived stress [29]. A systematic review of studies on physical activity and mindfulness found that there is a positive relationship between engaging in physical activity and trait mindfulness [30]. Schneider et al. [31] emphasize that activities aimed at the development of mindfulness are more effective when some form of physical activity is undertaken simultaneously. Similar conclusions are indicated by Remskar et al. [32] in a review of studies examining the effectiveness of physical activity combined with mindfulness training in reducing stress levels. Researchers showed that a combination of such interventions was more effective for lowering stress levels than either physical activity or mindfulness practice alone [32]. Individuals who undertook both interventions also scored lower on the Perceived Stress Scale (PSS-10) than those who had not undertaken any activity [32]. Schneider et al. [31] point out that when it comes to the development of mindfulness, the practice of yoga will be more effective than other forms of physical activity because it is particularly process oriented. Although yoga and physical exercise share similarities, they also demonstrate significant differences, with yoga providing greater health benefits due to its focus on breath regulation, mindfulness, and maintaining postures [33].

Having originated in ancient Indian culture, today, yoga most often takes the form of physical activity. Yoga is associated with the practice of yogic postures (asana), breathing techniques (pranayama), meditation techniques (pratyahara, dharana, dhyana, and samadhi), and ethical teachings (yamas and niyamas) [34,35,36]. Yoga is an ancient practice that has evolved into various forms and styles, each with unique components and benefits [37,38,39,40,41]. Yoga encompasses a variety of practices, each with unique benefits for physical, mental, and emotional well-being, including such types and styles as Vinyasa yoga (the most athletic yoga style), Hatha yoga (mainly focused on physical postures of yoga), Iyengar yoga (focuses on alignment as well as detailed and precise movements), Kundalini yoga (focuses on spiritual and physical parts equally by releasing the kundalini energy from the lower spine), Ashtanga yoga (involves a very physically demanding sequence of postures), Bikram yoga (includes a series of 26 basic postures, with each one performed twice, in a sauna-like room—typically set to 105 degrees and 40% humidity), Yin yoga (a slow-paced style of yoga with seated postures, with a meditative practice aimed to find inner peace), Restorative yoga (focuses on body relaxation, to cleanse and free the mind), Prenatal yoga (adapted to women in every trimester of pregnancy), Anusara yoga (a version of hatha yoga, focusing on alignment, with more emphasis on the mind–body–heart connection), and Jivamukti yoga (vinyasa-flow-style classes infused with Hindu spiritual teachings, emphasizing connection to Earth as a living being and vegetarian philosophy) [42]. Hatha Yoga is the most commonly practiced form, comprising specific active and restorative physical postures, breath control, and meditation, that emphasizes stretching exercises, improved physical postures, and concentration techniques [34,40].

The proposed mechanisms of yoga on stress, physical health, and emotional well-being comprise increasing psychological resources, such as mindfulness, positive affect, self-compassion, body consciousness, self-transcendence, spiritual peace, and social connectedness [43,44]. Indeed, studies indicate that people who practice yoga have higher levels of mindfulness, better quality of life, and lower levels of depression, anxiety, and stress [45,46]. Notably, yoga as a form of physical activity is more often chosen by women than men [41,47]. A nationally representative survey in the U.S.A. [48] showed that lifetime yoga practitioners were more likely to be female and younger. Additionally, the survey study [49] showed that among 36 million yoga practitioners in the U.S., 72% of them were women. Since yoga practice is more representative of women than men, a female sample will be a target in this study.

### The Present Study Objectives

Questions arise as to whether a sufficiently frequent yoga practice would be positively associated with trait mindfulness and, thus, lower perceived stress levels. This gap in the literature is filled in the present study. For the first time, to the best of our knowledge, the yoga practice time will be considered in its association with perceived stress and mindfulness traits in this study. Some previous studies showed that a high degree of yoga involvement is related to better mental health and well-being, along with lower levels of distress and depression [47,50]. However, yoga involvement was assessed in previous studies using a self-report questionnaire, the Yoga Immersion Scale [47,50]. In contrast, we would like to use a measure of time spent practicing yoga based on WHO recommendations [28]. We hypothesize that those women who practice yoga, according to the World Health Organization [28] recommendations on physical activity, would score higher on trait mindfulness and lower in perceived stress levels than women who practice yoga less than 150 min per week and those engaged in other forms of physical activity or people who are physically inactive.

In addition, the study aims to investigate the relationship between perceived stress and trait mindfulness. According to previously cited research [7,10,19,20], a negative relationship between perceived stress and trait mindfulness is anticipated in this study. Research [51] has shown that participating in physical activity and mindfulness meditation can reduce the stressful response to the COVID-19 pandemic. Goldstein et al. [52] have shown that mindfulness and exercise training have similar mechanisms that can improve adaptive responses to stress. Demarzo et al. [53] proposed the hypothesized role of dispositional mindfulness as a mediator of the effects of exercise training on cardiovascular responses to stress. However, to the best of our knowledge, this theoretical mediation model [53] has never been validated. Based on these theoretical assumptions [53], for the first time, both physical activity and yoga practice will be tested via dispositional mindfulness in a mediation model to verify the indirect effect of physical activity and yoga practice on perceived stress. We will additionally examine whether physical training alone or supported by yoga techniques will constitute a significant predictor of both trait mindfulness and perceived stress.

Therefore, this study aimed to verify the following assumptions:

**Q1.**  *Are there any differences in the levels of trait mindfulness and perceived stress between women who practice yoga and those who do not, considering their level of physical activity?*

**H1.**  *Women who practice yoga at least 150 min a week [28] score higher in trait mindfulness [31,32,47] and lower in perceived stress [31,32,45,46,50] compared to women engaged in physical activity and those who are physically inactive.*

**Q2.**  *Is there a relationship between trait mindfulness and perceived stress among women who practice yoga and are physically active?*

**H2.**  *Trait mindfulness is negatively associated with perceived levels of stress [7,10,19,20]*.

**Q3.**  *Is the indirect effect of physical activity and yoga practice on perceived stress mediated by the trait of mindfulness?*

**H3.**  *The indirect effect of physical activity and yoga practice on perceived stress is explained through trait mindfulness [53].*

## 2. Materials and Methods

### 2.1. Procedure

This study had the form of an online survey conducted between 25 June 2023, and 4 October 2023. A link to the survey was shared on the social media platforms Facebook and Instagram, inviting women, both practitioners and non-practitioners of yoga, to participate. The respondents were informed about the purpose and course of the study, their anonymity was ensured, and they were given the option to stop completing the survey at any time. Having given consent to participate in the study, the respondents proceeded to the first demographic section, which consisted of questions on age, education, place of residence, and whether they practice yoga. Depending on their answer to the last question, the participants moved on to the next section accordingly. Women who declared that they practiced yoga completed the yoga section (exercise frequency per week, duration of one session, type of yoga practiced). Women who asserted they were not practicing yoga answered questions related to physical activity (frequency of activity per week, minutes spent on one exercise session, type of activity). Questions about the type of yoga or physical activity allowed multiple answer options. In the next step, respondents were asked to complete two questionnaires. The Mindful Attention Awareness Scale (MAAS) was used to measure trait mindfulness, and the Perceived Stress Scale (PSS-10) was used to measure stress.

### 2.2. Participants

The study included 201 women, of which 96 were yoga practitioners and 105 were non-practitioners of yoga. The average age of all participants was 36 years (*M* = 36.19, *SD* = 11.64); the youngest respondent was 18 and the oldest was 72. Detailed demographic data are shown in Table 1. Women practicing yoga did not differ from those not practicing in minutes spent on practicing yoga or physical activity per week [*t*(199) = −1.71, *p* = 0.089, Cohen’s *d* = 0.24], place of residence [χ^2^(4) = 3.84, *p* = 0.429, Cramer’s V = 0.14], and education [χ^2^(4) = 7.76, *p* = 0.101, Cramer’s V = 0.20]. However, yoga practitioners were significantly older than those not practicing yoga [*t*(199) = −3.98, *p* < 0.001, Cohen’s *d* = 0.56]. Among the various types of yoga, the prevailing types were Vinyasa yoga (*n* = 44, 45.83%), Hatha yoga (*n* = 36, 37.5%), Yin yoga (*n* = 26, 27.08%), no specific type of yoga (*n* = 26, 27.08%), Ashtanga yoga (*n* = 23, 23.96%), Iyengar yoga (*n* = 22, 22.92%), yoga Nidra (*n* = 17, 17.71%), power yoga (*n* = 12, 12.5%), Aerial yoga (*n* = 3, 3.13%), integral fascial yoga (*n* = 2, 2.08%), Kundalini (*n* = 2, 2.08%), Sivananda yoga (*n* = 1.04, 1.04%), sup yoga (*n* = 1, 1.04%), Sri Sri yoga (*n* = 1, 1.04%), functional yoga (*n* = 1, 1.04%), and balance board yoga (*n* = 1, 1.04%). Among women not practicing yoga, the following sports disciplines and recreational physical activities were represented: walking (*n* = 75, 71.43%), cycling (*n* = 36, 34.29%), gym workout (*n* = 21, 20.00%), swimming (*n* = 13, 12.38%), dancing (*n* = 13, 12.38%), running (*n* = 10, 9.52%), no physical activity (*n* = 8, 7.67%), fitness (*n* = 7, 6.67%), Nordic walking (*n* = 6, 5.71%), team sport (*n* = 4, 3.81%), horse riding (*n* = 4, 3.81%), tennis (*n* = 2, 1.9%), Pilates (*n* = 2, 1.9%), stretching (*n* = 2, 1.9%), trekking (*n* = 2, 1.9%), rehabilitation exercises (*n* = 2, 1.9%), martial arts (*n* = 1, 0.95%), and Tibetan rites (*n* = 1, 0.95%).

### 2.3. Measures

#### 2.3.1. Trait Mindfulness

The Mindful Attention Awareness Scale (MAAS) is a tool designed by Brown and Ryan [7] to assess trait mindfulness. This study uses the Polish adaptation of the MAAS [8], consisting of 15 items, with a 6-point response scale (from 1 = “almost always” to 6 = “almost never”) to assess how often a respondent experiences the situations indicated in the statements. The total score is the average of all responses in the MAAS. The higher the score, the higher is the level of trait mindfulness. The reliability (Cronbach’s α) in the Polish validation study [8] ranged between 0.81 and 0.85, while in this study sample, it was 0.87.

#### 2.3.2. Perceived Stress

The PSS-10 scale by Cohen et al. [54], used to assess the level of stress over the past month, was adapted to the Polish language by Juczyński and Ogińska-Bulik [55]. The PSS-10 includes ten items, and respondents answer on a 5-point scale from 0 (“never”) to 4 (“almost always”) how often they perceived the given stress response during the last month. The higher the sum of all the scores, the higher is the level of perceived stress. The reliability coefficient in the present study was Cronbach’s α = 0.91.

#### 2.3.3. Demographics

Demographic questions concerned age (years), place of residence (Village, City up to 50 thousand inhabitants, City from 50 thousand up to 150 thousand inhabitants, City from 150 thousand up to 500 thousand inhabitants, City with over 500 thousand inhabitants), education (Primary, Vocational, Lower secondary, Secondary, Undergraduate, Graduate), and whether they practice yoga (Yes, No). Questions strictly related to yoga practice include the frequency of yoga practice per week (1–7 days; I don’t practice yoga systematically), the duration of one session (open question—in minutes), and the type of yoga practiced (Vinyasa yoga, Hatha yoga, Yin yoga, Ashtanga yoga Power yoga, Iyengar yoga, Aerial yoga, Acro yoga, Yoga Nidra, I don’t practice a specific type of yoga, Other—open question). Items related to physical activity concerned the frequency of physical activity per week (1–7 days; I don’t do physical activity systematically), duration of one training session (open question—in minutes), and the type of physical activity practiced (Walking, Cycling, Gym workout, Swimming, Dancing, Running, Nordic walking, Team sport, Martial arts, I don’t do any physical activity, Other—open question).

### 2.4. Statistical Analysis

Descriptive statistics were performed preliminarily, including the mean (*M*), standard deviation (*SD*), median (*Mdn*.), skewness, and kurtosis, to assess the parametric properties of trait mindfulness (MAAS) and perceived stress (PSS-10). Since the sample size was quite large (*N* = 201), and the skewness and kurtosis ranged between plus/minus 1, we decided to use parametric tests for further statistical analyses. Four groups were compared taking into account whether the participant practiced yoga (yes or no) and the time spent on physical activity or yoga practicing (minimum 150 min per week or less than 150 min per week): no active and no yoga (NANY, coded as 0, *n* = 69), physically active and no yoga (PANY, coded as 1, *n* = 36), no active and yoga active (NAYA, coded as 2, *n* = 50), and physically active and yoga active (PAYA, coded as 3, *n* = 46). A one-way analysis of variance (ANOVA) was performed to examine the effect of the group on trait mindfulness and perceived stress. The Bonferroni post hoc test was used to check for significant intergroup differences, and the partial eta-square test ($\eta_{p}^{2}$) was used to assess the effect size. The association between trait mindfulness and perceived stress was examined using Pearson’s correlation. Furthermore, G.L.M. mediation analysis was performed to test the indirect effect of activity type (yoga and physical activity) on perceived stress via trait mindfulness [56]. A bias-corrected bootstrap (B.C.B., with 5000 replications) was computed to assess the confidence intervals (95% CIs). All analyses were conducted using JAMOVI, ver. 2.3.28 [57]. 

## 3. Results

### 3.1. Differences in Trait Mindfulness and Perceived Stress among Women Practicing Yoga and Physically Active

**Figure 1:**
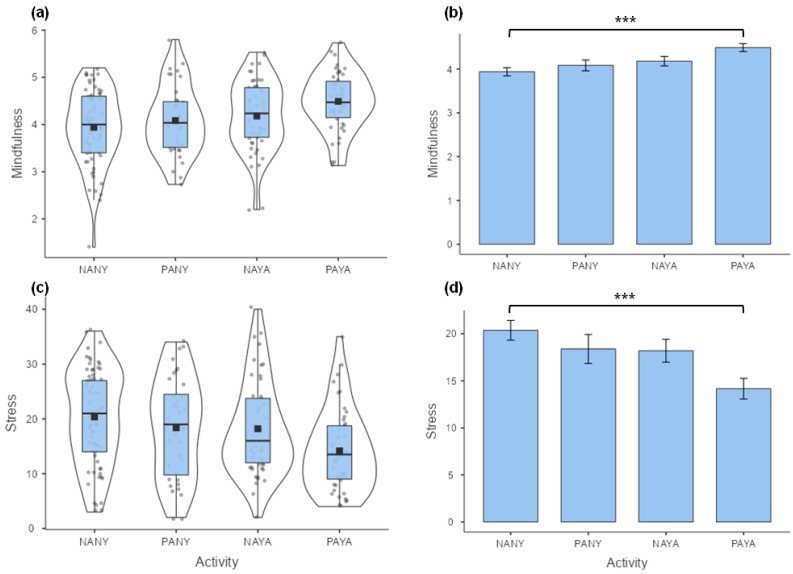
The distribution of scores (**a**,**c**) and mean results (**b**,**d**) for trait mindfulness (**a**,**b**) and perceived stress (**c**,**d**) in groups representing four types of activity in women. NANY = no active and no yoga, PANY = physically active and no yoga, NAYA = no active and yoga active, PAYA = physically active and yoga active. The dots in the figure represent the individual results of the participants. Error bars are standard errors (*SE*). *** *p* < 0.001.

The distribution of scores and mean differences between samples of women (NANY, PANY, NAYA, and PAYA) in trait mindfulness and perceived stress are presented in Figure 1. The one-way ANOVA showed that the groups of women differ in trait mindfulness, *F* (3, 197) = 5.34, *p* = 0.001, $\eta_{p}^{2}$ = 0.08 (Figure 1a,b). In particular, the Bonferroni post-test showed that women practicing yoga for at least 150 min per week scored higher in trait mindfulness (PAYA) than non-physically active women (NANY) who did not practice yoga, Δ*M* = −0.55, *S.E.* = 0.14, *t* = −3.95, *p* < 0.001, Cohen’s *d* = −0.75. The group effect was also significant for perceived stress level, *F* (3, 197) = 4.92, *p* = 0.003, $\eta_{p}^{2}$ = 0.07 (Figure 1c,d). The sample of women who practice yoga for a minimum of 150 min per week showed significantly lower perceived stress levels than the non-physically active participants who did not practice yoga, Δ*M* = 6.19, *S.E.* = 1.62, *t* = 3.83, *p* < 0.001, Cohen’s *d* = 0.73.

### 3.2. Associations between Trait Mindfulness and Perceived Stress in Women Who Practice Yoga and Are Engaged in Physical Activity

**Figure 2:**
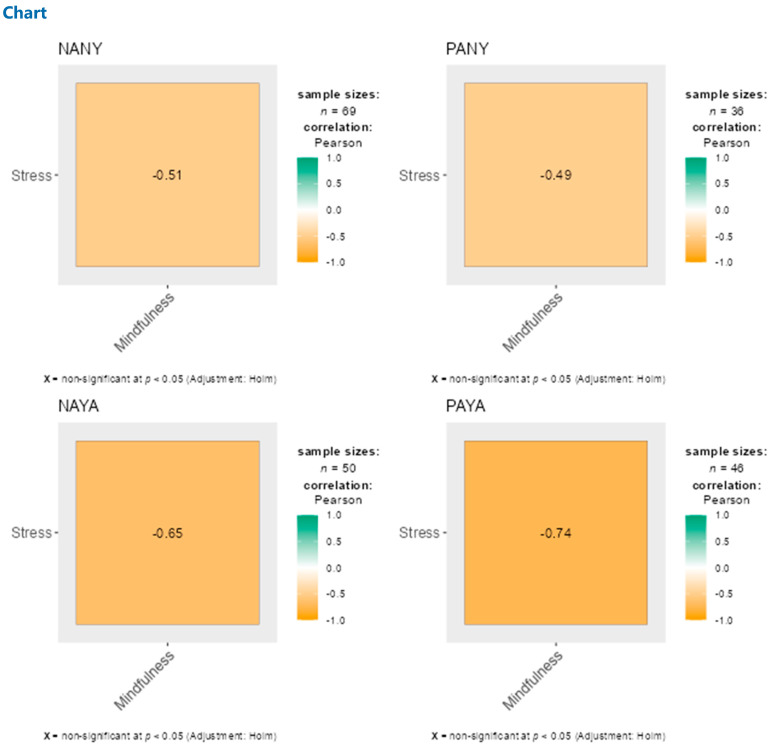
Pearson’s correlation between trait mindfulness and perceived stress in particular groups, depending on the type of activity (Yoga and physical activity). NANY = no active and no yoga, PANY = physically active and no yoga, NAYA = no active and yoga active, PAYA = physically active and yoga active.

**Figure 3:**
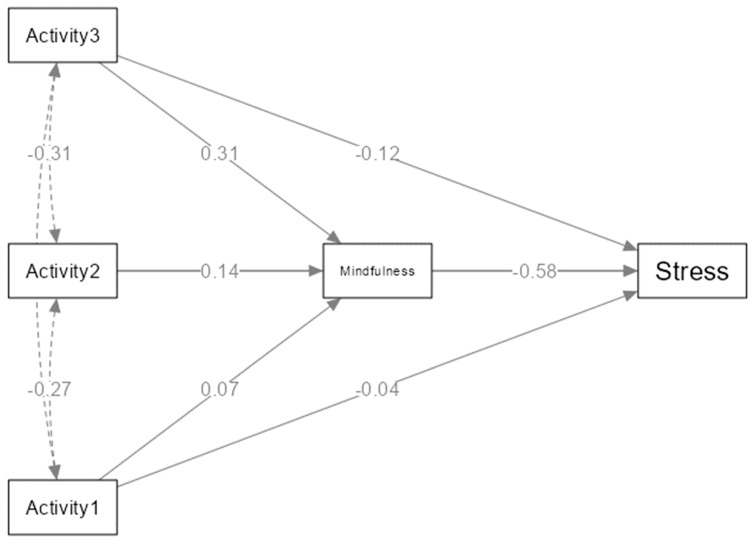
Mediation model for the indirect effect of activity type on perceived stress via trait mindfulness. Numbers represent standardized regression estimates (β). For the variable Activity, the contrasts are: Activity1 = PANY–NANY, Activity2 = NAYA–NANY, Activity3 = PAYA–NANY, where NANY = no active and no yoga, PANY = physically active and no yoga, NAYA = no active and yoga active, PAYA = physically active and yoga active.

In the total sample, trait mindfulness is negatively related to perceived stress, *r* = −0.61. The correlations for the separate groups ranged between −0.49 and −0.74 (see Figure 2 for more details). A mediation analysis was conducted via trait mindfulness to examine the indirect effect of activity type on perceived stress levels (Table 2 and Figure 3). A multi-categorical type of activity as a predictor variable was included in the mediation model, with not physically active and not practicing yoga women as a reference group. Age was added to the mediation model as a confounder covariate. The total effect on perceived stress reduction was significant only in those participants who practiced yoga for at least 150 min per week. Additionally, higher trait mindfulness was predicted only in the same sample of active yoga practitioners. Consequently, the indirect effect of trait mindfulness was found in the group of women who practice yoga in a manner that is consistent with the WHO’s recommendation (minimum 150 min per week).

## 4. Discussion

This study aimed to examine differences in the levels of trait mindfulness and perceived stress between women practicing yoga and people engaged in other forms of physical activity, taking into account the recommended WHO duration of regular exercise as a minimum of 150 min per week [28,41]. In addition, we examined the role of yoga practice and physical activity in reducing perceived stress levels by means of dispositional mindfulness to verify the mediation model hypothesized by Demarzo et al. [53]. Previous research also indicates that physical activity has a positive effect on improving the mental health of individuals by means of developing trait mindfulness and reducing perceived stress levels [29,30,51,52]. Among the various types of physical activity, yoga seems exceptionally potent for developing mindfulness and lowering perceived stress, as it involves not only physical exercise but also meditation and breathing exercises [31,45,47,58].

Trait mindfulness plays an essential role in reducing perceived stress levels, and interventions aimed at its development prove to be more effective when physical activity is undertaken at the same time [7,10,19,20,32]. The analysis of intergroup differences showed that women who practice yoga and are adequately physically active have significantly higher levels of trait mindfulness and lower levels of perceived stress than women who do not practice yoga and are not physically active, which partially supports our hypothesis. Women who are adequately physically active and choose to practice yoga presumably benefit not only from the physical activity itself but also from various forms of meditation and breathing techniques. The lack of significant differences in the other groups may be due to the fact that physical activity alone or a yoga practice of less than 150 min per week may, to some extent, develop mindfulness and reduce perceived stress. In accordance with the hypothesis and results of previous research, this study found a negative association between trait mindfulness and perceived stress. More mindful individuals may be better at accessing their resources as well as assessing reality and evaluating possible danger. Their acceptance of stressful experiences makes mindful individuals exhibit greater cognitive and behavioral flexibility, as well as the ability to self-regulate, which may contribute to their lower levels of perceived stress. The relationship between trait mindfulness and perceived stress was found to be strongest in a group of women who practice yoga and are, at the same time, adequately physically active.

In order to examine the role yoga practice and physical activity play in reducing perceived stress levels via trait mindfulness, a mediation analysis was performed. The theoretical mediation model proposed by Demarzo et al. [53] is partially confirmed in the study since the significant mediation effect is presented only for women practicing yoga at least 150 min a week. The meditational aspect of regular yoga practice can effectively improve mindfulness skills and, in turn, significantly reduce perceived stress levels, which seems to be consistent with previous studies [51,52]. However, the findings suggest that only sufficient yoga practice can reduce perceived stress via improving mindfulness skills, whereas practice less than 150 min per week or engagement in other physical activity does not affect trait mindfulness. The results of this study seem to be in line with other findings, indicating that only an adequate level of exercise can improve health and well-being [7,10,19,20,32]. In contrast to previous research, the present study has shown that undertaking other forms of physical activity than yoga, even above 150 min per week, does not contribute to perceived stress reduction via trait mindfulness. This study highlights the unique aspects of yoga practice, as well as the significance of practicing it in accordance with WHO [28] recommendations for physical activity to maintain well-being.

### Limitations and Further Research

This study had certain limitations. Firstly, the study group was limited to only women who practice yoga and those who do not practice yoga, and the group was too small to be considered representative. Because the study did not include men, the results cannot be generalized to men who practice yoga and are physically active. Yoga was measured in this study as a single construct, although it encompasses many different practices with varying physical demands, and as such, the results require replication for subgroups practicing different types of yoga. The study results cannot be generalized to objective measures of stress and other questionnaires measuring chronic and acute stress responses. It needs to be taken into account that the study conducted was correlational and cross-sectional, which does not allow for causal conclusions. Despite its limitations, the study findings are interesting and worth exploring in further research. A question that remains to be investigated in the future is the impact of the level of engagement in one’s yoga practice, which may be equally crucial for the development of mindfulness and perceived stress reduction among female yoga practitioners. Trait mindfulness and perceived stress were assessed using self-report questionnaires. Future research aimed at drawing causal relationships would require a series of longitudinal and experimental studies to be conducted using various methods of stress and mindfulness measurement (e.g., psychophysiological stress measurement and therapy based on mindfulness techniques). Remskar et al. [32] noted that combining physical activity with mindfulness development training is more effective for stress reduction. It would be interesting to compare such interventions with undertaking only a yoga practice that includes elements of both physical activity and mindfulness development.

Another critical research direction would be to explore the relationship between yoga and the ongoing processes of globalization within the Polish context. Similar to the work of Maddox and Cooley [59] in examining the history of Los Angeles, it would be valuable to investigate the impact of the development and spread of yoga culture on Polish society. Additionally, it seems important to consider the availability of sports infrastructure for engaging in physical activities. Glebova and Desbordes [60] associate the development of smart cities with increased access to sports infrastructure. Therefore, future research should consider the influence of sports infrastructure accessibility on the examined variables.

In addition, it would be worthwhile to increase the number of study subjects and expand the study group to include men and people with other gender identities. Furthermore, age should be better distributed and more balanced in future study samples. The study group was diverse in terms of both types of physical activity and types of yoga. Future studies could compare selected types of activity with a homogeneous group in terms of the type of yoga. Another compelling line of research could include performing clinical trials to explore the benefits of practicing yoga.

## 5. Conclusions

The present study demonstrates a novel finding regarding the benefits of yoga practice. It was previously known that yoga practitioners are more mindful and less stressed [45,46]. This study showed that yoga training is associated with the heightened trait of mindfulness and reduced perceived stress levels, but only if it is practiced for a minimum of 150 min per week, which is in line with the WHO recommendations [28]. Moreover, research showed that undertaking other forms of physical activity does not predict perceived stress reduction via mindfulness traits. Therefore, it can be valuable to promote the practice of yoga, especially among women who experience stress and do not engage in any form of physical activity. Still, it is essential to point out the importance of a sufficient frequency of the practice.

This study found differences in mindfulness traits and perceived stress levels between women who practice yoga and are physically active and women who do not engage in any physical activity. Moreover, yoga, which is practiced according to the WHO recommendations, has been related to increased mindfulness traits and decreased perceived stress levels. This study carries important implications for women who practice yoga and for people who promote the benefits of practicing yoga. It is crucial to practice yoga a minimum of 150 min a week to significantly and effectively reduce stress levels. In addition, the study may provide a basis for implementing preventive measures among women experiencing stress.

## Figures and Tables

**Table 1:** Participant characteristics.

Variable	Category	Total Sample (*N* = 201)		Practicing Yoga (*n* = 96)		Not Practicing Yoga (*n* = 105)	
		*M*/*n*	*SD*/%	*M*/*n*	*SD*/%	*M*/*n*	*SD*/%
Age		36.19	11.65	39.49	11.44	33.18	11.05
Residence	Village	41	20.4	17	17.7	24	22.86
	City < 50,000 inhabitants	28	13.93	14	14.58	14	13.33
	City 50,000 < 150,000 inhabitants	42	20.9	18	18.75	24	22.86
	City 150,000 < 500,000 inhabitants	31	15.42	13	13.54	18	17.14
	City > 500,000 inhabitants	59	29.35	34	35.42	25	23.81
Education	Lower secondary	1	0.5	0	0	1	0.95
	Vocational	3	1.49	1	1.04	2	1.9
	Secondary	34	16.92	10	10.42	24	22.86
	Undergraduate	45	22.39	21	21.86	24	22.86
	Graduate	118	58.71	64	66.67	54	51.43
Minutes of exercise per week		142.1	128.18	127.42	146.14	158.18	107.86

**Table 2:** Mediation effects: indirect, direct, and total.

Type	Effect	*B*	*SE*	95% BCB CI		β	*z*	*p*
				LL	UL			
Indirect	Activity1 ⇒ Mindfulness ⇒ Stress	−0.98	1.05	−3.15	0.98	−0.04	−0.93	0. 350
	Activity2 ⇒ Mindfulness ⇒ Stress	−1.63	0. 97	−3.63	0. 27	−0.08	−1.68	0. 093
	Activity3 ⇒ Mindfulness ⇒ Stress	−3.72	0.93	−5.62	−1.97	−0.18	−4.00	<0.001
Component	Activity1 ⇒ Mindfulness	0.15	0.15	−0.15	0.45	0.07	0. 95	0. 344
	Activity2 ⇒ Mindfulness	0.24	0.14	−0.04	0. 53	0.14	1. 69	0. 092
	Activity3 ⇒ Mindfulness	0.55	0.13	0.29	0.81	0.31	4. 21	<0.001
	Mindfulness ⇒ Stress	−6.76	0. 68	−8.05	−5.41	−0.58	−9.99	<0.001
Direct	Activity1 ⇒ Stress	−1.00	1. 64	−4.22	2. 19	−0.04	−0.61	0. 545
	Activity2 ⇒ Stress	−0.54	1. 35	−3.22	2.15	−0.03	−0.40	0. 692
	Activity3 ⇒ Stress	−2.47	1. 29	−5.02	0. 02	−0.12	−1.91	0. 056
Total	Activity1 ⇒ Stress	−1.97	1. 84	−5.54	1. 76	−0.09	−1.07	0. 285
	Activity2 ⇒ Stress	−2.16	1. 58	−5.16	0.93	−0.11	−1.37	0. 170
	Activity3 ⇒ Stress	−6.19	1. 53	−9.12	−3.04	−0.30	−4.03	<0.001

Note: Confidence intervals are computed using the bias-corrected bootstrap method (BCB CI). Betas (β) are completely standardized effect sizes.

## Data Availability

The data reported in this research are available on request from the corresponding author.
